# International Medical Congress

**Published:** 1874-09-01

**Authors:** 


### INTERNATIONAL MEDICAL CONGRESS.

WE have been requested to call the attention of the profession to the fact that the fourth session of the "International Periodical Congress for the Advancement of the Medical Sciences," will be held in the city of Brussels, Belgium, on the 6th of September, 1875. The circular issued by Prof. E. Warlomont, of the Royal Academy of Medicine, of Belgium, General Secretary of the Congress, gives in full the details of the proposed re-union, which will continue for one week. Delegates from Medical Associations, national and foreign, will be admitted by forwarding their credentials to the General Secretary, and will have the exclusive privilege of taking part in the discussions.

Members of the Congress will be assigned to five sections: the first, on Medicine, Surgery and Obstetrics; the second, on Military Surgery; the third, on Hygiene; the fourth, on Ophthalmology; and the fifth, on Pharmacology: each member being assigned to that section which he shall designate, and to more than one if it shall be so desired.

Those who are to present reports to the sections, will be designated by the committee appointed for that purpose; but other reports and communications will be received, not announced in the special order, the subjects of which are germane to the field assigned to each section. The definite conclusions adopted by each section will be submitted to the General Assembly by special reporters, selected by the former from among their number.

Those who desire to present communications on subjects not included in the order of business, must apply for permission to do so, at least one month before the session of the Congress, to the General Secretary. Permissions will be obtainable from the Committee on Organization, which will decide, also, as to the order of such extrinsic business.

Each speaker will be limited to twenty minutes in the discussion of a single question, but this limitation will not apply to the reporters of the Congress. All papers read before the Congress will be placed at its disposal, and, after its session, the Committee on Organization will decide as to the partial, total or non-insertion of such papers in its published transactions.

Although the French will be the language employed in the discussions of the Congress, members will be permitted to express themselves, if desired, in other languages. If it be considered expedient, interpreters will be employed to translate on the spot the communications addressed to the Assembly in such other languages.

The above is a brief summary of the facts represented in Prof. Warlomont's circular, which we thought might be of interest to the readers of the EXAMINER. The editors will be pleased to give any further information in regard to the Congress which may be sought by those who conclude to take a special interest in its transactions.

Communications to the General Secretary may be addressed to "M. le Dr. Warlomont, 152 Rue Royale, á Bruxelles, en Belgique."

